# Sequential Portal Vein–Hepatic Vein Embolization: Progress Yet Unaccounted Pitfalls

**DOI:** 10.1002/ags3.70148

**Published:** 2025-12-07

**Authors:** Syeda Rabiah Shahid, Ahmad Furqan Anjum

**Affiliations:** ^1^ Shaikh Khalifa Bin Zayed Al‐Nahyan Medical and Dental College Lahore Punjab Pakistan; ^2^ Shaikh Zayed Postgraduate Medical Institute (SZPGMI) Lahore Punjab Pakistan

Dear Editor,

1

We read with great interest the article by Lai et al., providing valuable insights into sequential hepatic vein embolization for liver regeneration [1]. The authors should be commended for their contribution to advancing preoperative strategies that aim to expand feasible resection. However, we wish to highlight several concerns that may influence the interpretation and generalizability of the findings.

*First*ly, the study exclusively employed a single functional modality, the 99mTc‐GSA scintigraphy. The lack of standardized endpoints and universal validation of cutoff thresholds (GSA‐Rmax), limits generalizability of the results, especially in patients with liver cirrhosis. Moreover, introduction of conclusion bias in favor of a single imaging modality further prevents comparison with centers employing mebrofenin hepatobiliary scintigraphy (HBS) or EOB‐MRI [2]. *Secondly*, the outcomes were measured primarily up to hepatectomy, focusing on hypertrophy and short‐term function, while overlooking long‐term endpoints‐which defines the ultimate clinical utility of hypertrophy strategies‐including survival, recurrence, and disease‐free outcomes [3, 4]. *Moreover*, the tumor progression risk is not accounted for. The exclusion of patients experiencing progression introduces attrition bias and underestimates the risk of progression during waiting periods‐often reducing resectability and diminished oncological benefit [3]. *Fourth*, the variability in the embolization targets within the PVE–HVE group (RHV vs. RHV + IRHV) and the inconsistent time periods of recruitment, introduces variability and inconsistency. This heterogeneity, along with a lack of randomization, may confound results and overstate efficacy [4]. *Fifth*, the lack of generalizability of these findings of the trial conducted in a single high‐volume center, creates a steeper learning curve for smaller institutions. Sequential PVE–HVE requires multiple interventions, repeat anesthesia, imaging, and extended hospital stays [5], yet no cost‐effectiveness analysis was performed. Without weighing benefits against use of resources, feasibility in real‐world and resource‐limited settings remains uncertain.

Thus, the future studies should adopt standardized functional imaging protocols across centers, combining modalities for validation with an extended follow‐up period to include both late volumetric/functional regeneration (≥ 6–8 weeks) and long‐term oncological outcomes including survival, recurrence, and disease‐free intervals. Additionally, studies incorporating oncological endpoints as primary outcomes, including tumor progression rates, dropout from surgery, and time‐to‐resection, are warranted. This comprehensive approach will not only provide a more accurate and clinically relevant assessment of sequential PVE–HVE but also ensure a transparent evaluation of risks of disease progression alongside hypertrophy benefits. The health‐economic analyses and logistical feasibility studies alongside clinical outcomes are needed to guide adoption in real‐world practice. Future research should include multi‐center, randomized controlled trials or well‐designed prospective matched cohorts with standardized embolization protocols to assess reproducibility, reduce bias and provide a more realistic representation of outcomes across different healthcare environments.

In conclusion, while Lai et al. [1] provide valuable data on sequential PVE–HVE, the absence of standardized endpoints, limited follow‐up, exclusion of progression cases, methodological variability, and omission of cost considerations limits the strength and applicability of their conclusions (Figure 1). Addressing these gaps through prospective, multicenter studies with standardized protocols, comprehensive oncological follow‐up, and economic evaluations will be essential to defining the true role of sequential PVE–HVE in clinical practice.

**FIGURE 1:**
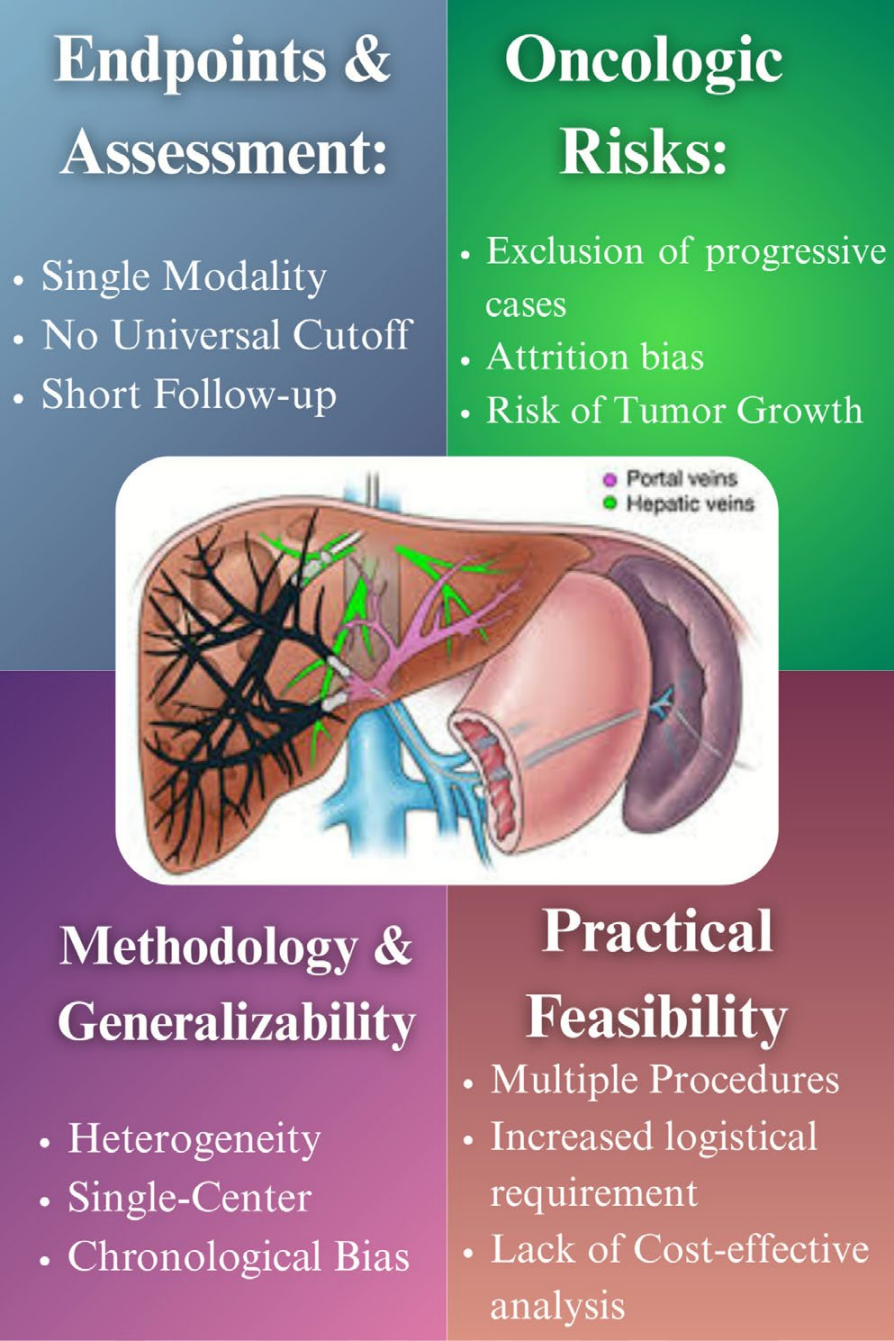
Central illustration of Sequential Portal Vein–Hepatic Vein Embolization (PVE–HVE), summarizing its progress, unaccounted pitfalls, and clinical limitations.

## Author Contributions

All the authors meet the ICMJE authorship criteria and have made significant and equal contributions to this manuscript. All authors approved the final version and agree to be accountable for all aspects of the work, ensuring the accuracy and integrity of the data and interpretation.

## Funding

The authors has nothing to report.

## Disclosure

*Guarantor Statement*: All authors have read and approved the final version of the manuscript. They take complete responsibility for the integrity and accuracy of the data being shared.

*Transparency Statement*: The Authors, affirm that this manuscript is an honest, accurate, and transparent account of the study being reported, that no important aspects of the study have been omitted, and that any discrepancies from the study as planned (and if relevant, registered) have been explained.

## Ethics Statement

The authors have nothing to report.

## Conflicts of Interest

The authors declare no conflicts of interest.

## Linked Articles

This article is linked to Lai et al. papers. To view this article, visit https://doi.org/10.1002/ags3.70085.

## Data Availability

Data sharing does not apply to this article as no datasets were generated during the current study; all data were sourced from published literature.
